# Influence of Carboxymethyl Cellulose on the Green Synthesis of Gold Nanoparticles Using *Gliricidia sepium* and *Petiveria alliacea* Extracts: Surface-Enhanced Raman Scattering Effect Evaluation

**DOI:** 10.1021/acsomega.3c03813

**Published:** 2023-11-29

**Authors:** Sindi Horta-Piñeres, M. Cortez-Valadez, Duber A. Avila, Jesús Eduardo Leal-Perez, Cesar Cutberto Leyva-Porras, Mario Flores-Acosta, Cesar O. Torres

**Affiliations:** † Laboratorio de Óptica e Informática, Universidad Popular del Cesar, Apdo. Postal, Valledupar, Cesar 200001, Colombia; ‡ Departamento de Investigación en Física, Universidad de Sonora, Apdo. Postal 5-88, Hermosillo, Sonora 83190, México; § CONACYT-Departamento de Investigación en Física, Universidad de Sonora, Apdo. Postal 5-88, Hermosillo, Sonora 83190, México; ∥ Centro de Investigaciones en Materiales Avanzados S.C. (CIMAV), Chihuahua 31136, México; ⊥ Facultad de Ingeniería Mochis, Universidad Autónoma de Sinaloa, Los Mochis, C.P. 81223, México

## Abstract

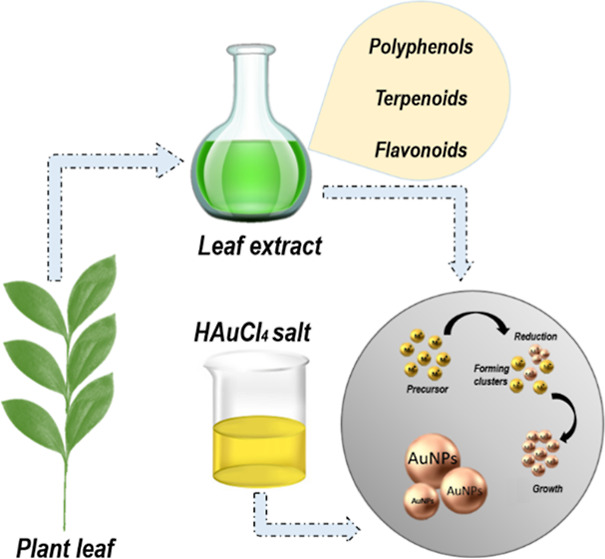

Gold nanoparticles (AuNPs) were synthesized and stabilized using ecological strategies: the extracts of the leaves of the plants *Gliricidia sepium* (GS) and *Petiveria alliacea* (PA) reduced the metallic Au ions to AuNPs. The AuNPs were analyzed as surface-enhanced Raman scattering (SERS) substrates for pyridoxine detection (vitamin B6). UV–vis spectroscopy was carried out to assess the stability of the AuNPs. As a result, absorption bands around 530 and 540 nm were obtained for AuNPs-PA and AuNPs-GS, respectively. Both cases associated it with localized surface plasmon resonance (LSPR). In the final stage of the synthesis, to stabilize the AuNPs, carboxymethyl cellulose (CMC) was added; however, LSPR bands do not exhibit bathochromic or hypsochromic shifts with the addition of CMC. Transmission electron microscopy (TEM) micrographs show relatively spherical morphologies; the particle diameters were detected around 7.7 and 12.7 nm for AuNPs-PA and AuNPs-GS, respectively. The nanomaterials were evaluated as SERS substrates on pyridoxine, revealing an intensification in the vibrational mode centered at 688 cm^–1^ associated with the pyridinic ring. Complementarily, different density functional theory functionals were included to obtain molecular descriptors on the Au_*n*_-cluster-pyridoxine interaction to study the SERS behavior.

## 1 Introduction

Within the diversity of nanomaterials, metallic nanoparticles (MNPs) have been recurrently studied for a range of applications based on their physicochemical and biological properties, which differ from their analogue in bulk and are strongly influenced by their metallic nature (gold, silver, and copper, among others), due to their size and morphology. Catalytic,^1^ sensing,^2,3^ cancer treatment,^4^ antimicrobial,^5^ fertilizer,^6^ and wastewater remediation applications^7,8^ have been reported for MNPs. To synthesize MNPs, physical, chemical, or biological methods are used; here, there are two techniques to synthesize nanoparticles: microorganism-mediated synthesis and plant-mediated synthesis.^9^ Plant-mediated synthesis, also known as biosynthesis or green synthesis, dates back to the 1990s and became known as “green chemistry”; in this synthesis, the reducing agent for metal ions extracts one or more parts of a plant.^3^ It has quickly become a growing research area and is viable for obtaining MNPs; it is fast and ecologically safe and has promising results.^10^ Reports show different extraction methods,^11^ but conventionally, the plant extract can be obtained by boiling the biomass inside a solvent (commonly water) and filtering the mixture. Countless varieties of plants and parts of plants, such as leaves, fruit, flowers, stems, and roots, have been used for the green synthesis of metallic nanoparticles. Within plant-mediated synthesis, the phytochemicals present in the extracts, such as phenolic components, glycosides, terpenoids, and alkaloids,^12−14^ are responsible during and after the synthesis for reducing metal ions and stabilizing the corresponding nanoparticles. However, although the ecological, economic, and biocompatible aspects represent the main advantage of this synthesis technique, the phytocomponents or their derivatives present in the final product degrade, and the MNPs agglomerate over time,^15^ precipitating and losing their nanometric nature. To cancel this effect, the chemical synthesis of MNPs uses stabilization elements such as formaldehyde, polyethylene glycol, and oleic acid, among others; however, they can cause harmful environmental effects. So, avoiding the aggregation of the molecules and the stability of the MNP colloids is a fundamental aspect to guarantee the applicability of the MNPs.^16^ Therefore, the molar concentration, solvent, proportions, and stabilizing ligands play a fundamental role. In green synthesis, to prevent the aggregation and sedimentation of MNPs, the aggregation must be totally or partially suppressed; that is, the repulsion forces between particles must compensate for the van der Waals forces. For this purpose, one can follow two strategies: steric stabilization and electrostatic stabilization.^17^ Steric stabilization involves using macromolecular “brushes” that surround or coat the nanoparticles, which prevent them from getting closer. This stabilization is very sensitive to the concentration of the stabilizing agents since, at low concentrations, the polymers interact with several particles simultaneously, inducing aggregation, and, when using high concentrations, the free polymers generate depletion forces between the particles, and aggregation may occur.^18^

On the other hand, electrostatic stabilization consists of the nonaggregation of the nanoparticles due to the potential barrier between the charges generated on the nanoparticle’s surface and those of the solution surrounding it. Electrostatic stabilization is a mechanism susceptible to modifying the charge distribution around the nanoparticle. According to the stabilization of MNPs, fluorides, carboxylates, polymers, and acids have been reported;^19−21^ however, these can modify the chemistry of the surface and the electrical, catalytic, and optical properties. Natural derivatives such as chitosan,^22^ hydroxypropylmethylcellulose,^23^ and other cellulose derivatives such as ethylcellulose^24,25^ and carboxymethylcellulose^26,27^ have also been used. The latter is mentioned as a reducing agent and stabilizer of MNPs^26,27^ and as a matrix to form metallic films^28^ for biomedical applications.^29,30^ Carboxymethyl cellulose or CMC is a linear-chain macromolecular polymer based on covalent bonds of d-glucopyranoses^24,31^ due to its hydrosolubility and biocompatibility properties. This research focuses on the use of CMC as a stabilizing and supporting agent for AuNPs and its use for pyridoxine (Pd) (vitamin B6) detection by surface-enhanced Raman scattering (SERS); it also highlights the biocompatibility of the SERS analysis with the green synthesis of MNPs, stimulating the promotion of additional biocompatible mechanisms integrated with this synthesis methodology for the conservation and applicability of colloidal nanoparticles.^32^

## 2 Materials and Methods

### 2.1 Preparation of Aqueous Extracts

The *Gliricidia sepium* (GS) and *Petiveria alliacea* (PA) plants were selected to prepare the aqueous extracts. First, the leaves of the GS and PA were carefully washed with deionized water. Next, 16 g of leaves, cut into small parts, was added in 240 mL of deionized water at 60 °C for 30 min, and then the preparations were filtered with a 65 g/m^2^ Boeco Germany filter paper and preserved until the synthesis of the AuNPs.

### 2.2 Preparation of the AuNPs

**Scheme 1:**
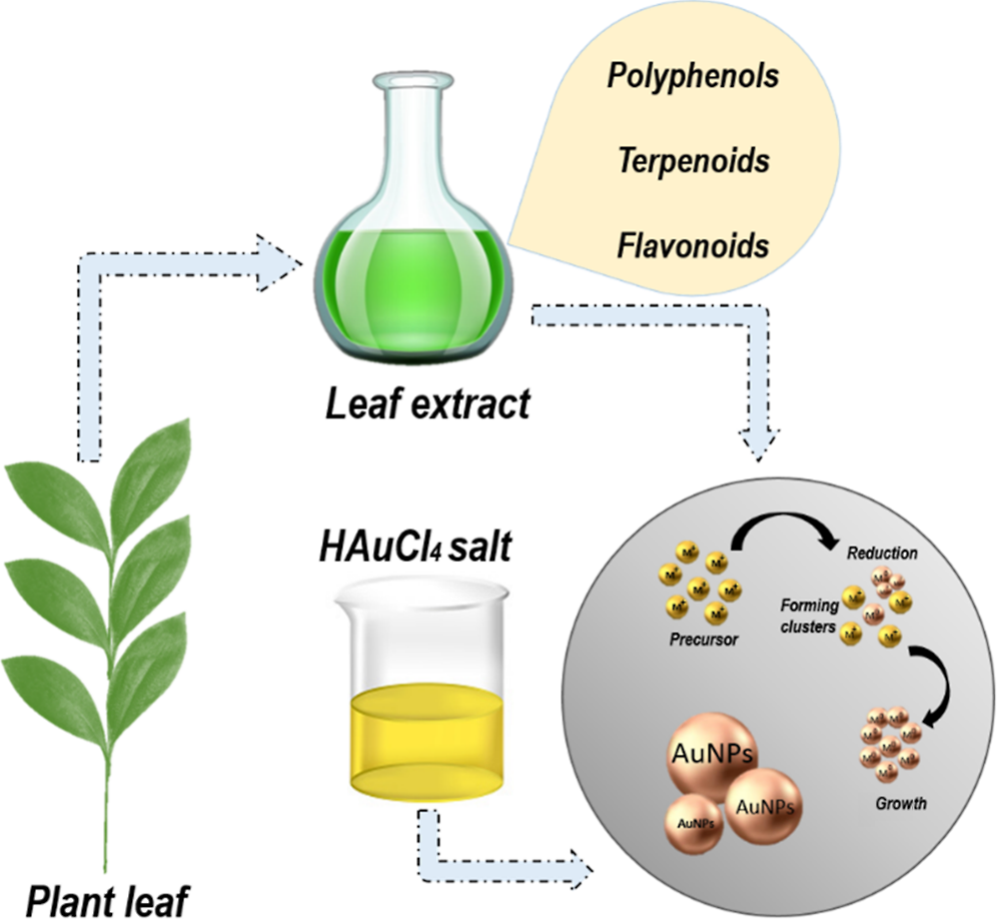
Steps in the Synthesis of AuNPs

HAuCl_4_ was prepared at 1 mM. First, in a beaker, 2 mL of aqueous extract in 15 mL of H_2_O was added; 2 mL of the precursor solution was added later, as shown in Scheme 1. The mixture was kept at 80 °C with magnetic stirring for up to 1 h. The scheme shows the synthesis of the AuNPs. During the synthesis, the formation of the AuNPs was confirmed by the color change from greenish yellow to pink at 5 min for the GS and approximately 25 min for the PA.

### 2.3 Stabilization with CMC

**Scheme 2:**
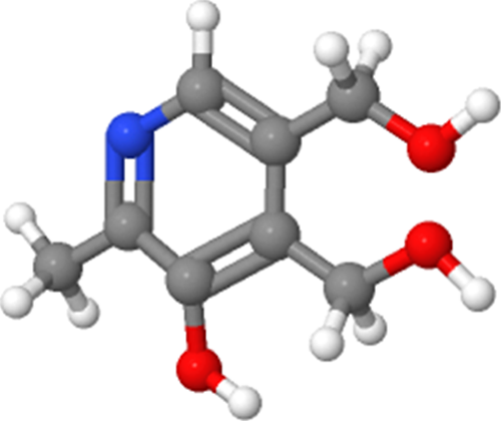
Molecular Structure of CMC

The CMC powder has a white or creamy-white color, is soluble in water but insoluble in oil and organic solvents, does not cause harm to the human body, is biodegradable, and plays an essential role in many industries, such as laundry, textile, paper, ceramics, paints, food, and medicine. CMC is also used as a viscosity additive, binder, and stabilizer and in packaging development.^33^ After synthesizing the AuNPs, 1 mL of CMC solution (8 mg/20 mL) was added and vigorously mixed with magnetic stirring, separated, and quickly placed in water at room temperature. Scheme 2 shows the molecular structure of CMC.

### 2.4 UV–Vis Absorption

The UV–vis spectra were measured with a VELAB 5100UV spectrophotometer in a wavelength range between 200 and 800 nm operated at a resolution of 1 nm. Samples were measured by transmission and diluted in deionized water. One μL of AuNPs was added to 2.5 mL of deionized water.

### 2.5 Transmission Electron Microscopy

The morphology, phase, and particle size were analyzed through transmission electron microscopy (TEM) with high-resolution TEM (HR-TEM) JEOL JEM-2200FS+Cs equipped with a spherical aberration corrector in a condenser lens and operated at 200 kV.

### 2.6 Raman Spectroscopy

Raman spectra were obtained with the LABram HR Evolution Raman spectrometer, Horiba (with AFM, AIST-NT coupled), with an excitation lambda of 780 nm, and selected commercial B6 vitamin (pyridoxine) for the SERS activity analysis of AuNPs-GS and AuNPs-PA.

### 2.7 Theoretical Methodology

Density functional theory (DFT) was used complementarily to obtain indications of the structural, electronic, and vibrational behavior. Specifically, LSDA (local spin density approximation), B3PW91 (Becke’s three parameters incorporating Perdew and Wang’s 1991), and HCTH (Hamprecht, Cohen, Tozer, Handy, and exchange–correlation hybrid functional) were used with the basis set LANL2DZ (Los Alamos National Laboratory 2 double-ζ), both included in the Gaussian 09 software.^34^ The gold clusters Au_6_, Au_8_, and Au_20_ were considered, representing a metallic surface, as well as the Pd molecule with the chemical formula C_8_H_11_NO_3_. The systems interacted until they obtained the minimum local energy, guaranteeing only positive frequencies in the predicted vibrational spectra. Some molecular descriptors made it possible to determine the affinity between the interacting systems, guaranteeing negative values in the adsorption energy and quantifying the degree of electron transfer. These calculations provided information about the magnitude and direction of charge transfer between the systems, helping to provide insights into the interactions and electronic effects involved.

## 3 Results and Discussion

**Figure 1:**
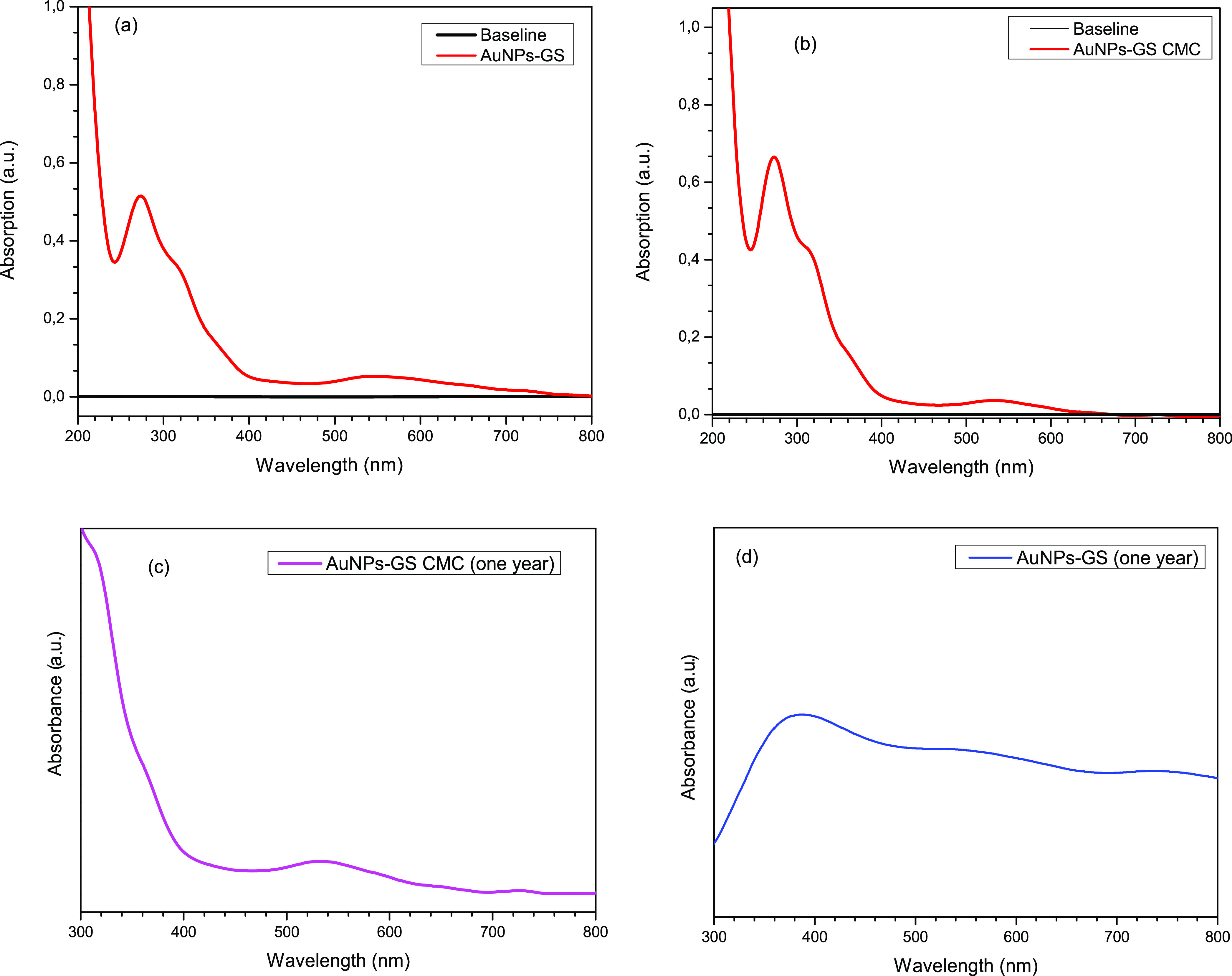
(a) UV–vis spectra of AuNPs-GS, (b) UV–vis spectra of AuNPs-GS stabilized with CMC, (c) UV–vis spectra of AuNPs-GS stabilized with CMC and obtained one year after synthesis, and (d) UV–vis spectra of AuNPs-GS without CMC one year after synthesis.

Figure 1 shows the UV–vis spectra of AuNP samples obtained in green synthesis with the GS plant extract without CMC (a) and with CMC (b). Both figures show an absorption band at 278 nm and a shoulder at 320 nm associated with the coumarin phytocomponent.^35^ The band around 550 nm is associated with the surface plasmon of the AuNPs-GS. Figure 1b shows the UV–vis spectrum of AuNPs-GS with CMC. This figure shows that CMC does not displace the optical absorption bands of the extract and the AuNPs-GS. To evaluate the stabilization and conservation of the AuNPs-GS colloid, Figure 1c,d shows the UV–vis spectra of the AuNPs-GS samples with CMC and without CMC, respectively, measured one year after the synthesis. Figure 1c shows that the LSPR band of AuNPs-GS is maintained, so the CMC prevented the aggregation and precipitation of the AuNPs-GS colloid. In contrast, Figure 1d shows the decay in the intensity of the LSPR band, indicating the aggregation of the AuNPs-GS colloid.

**Figure 2:**
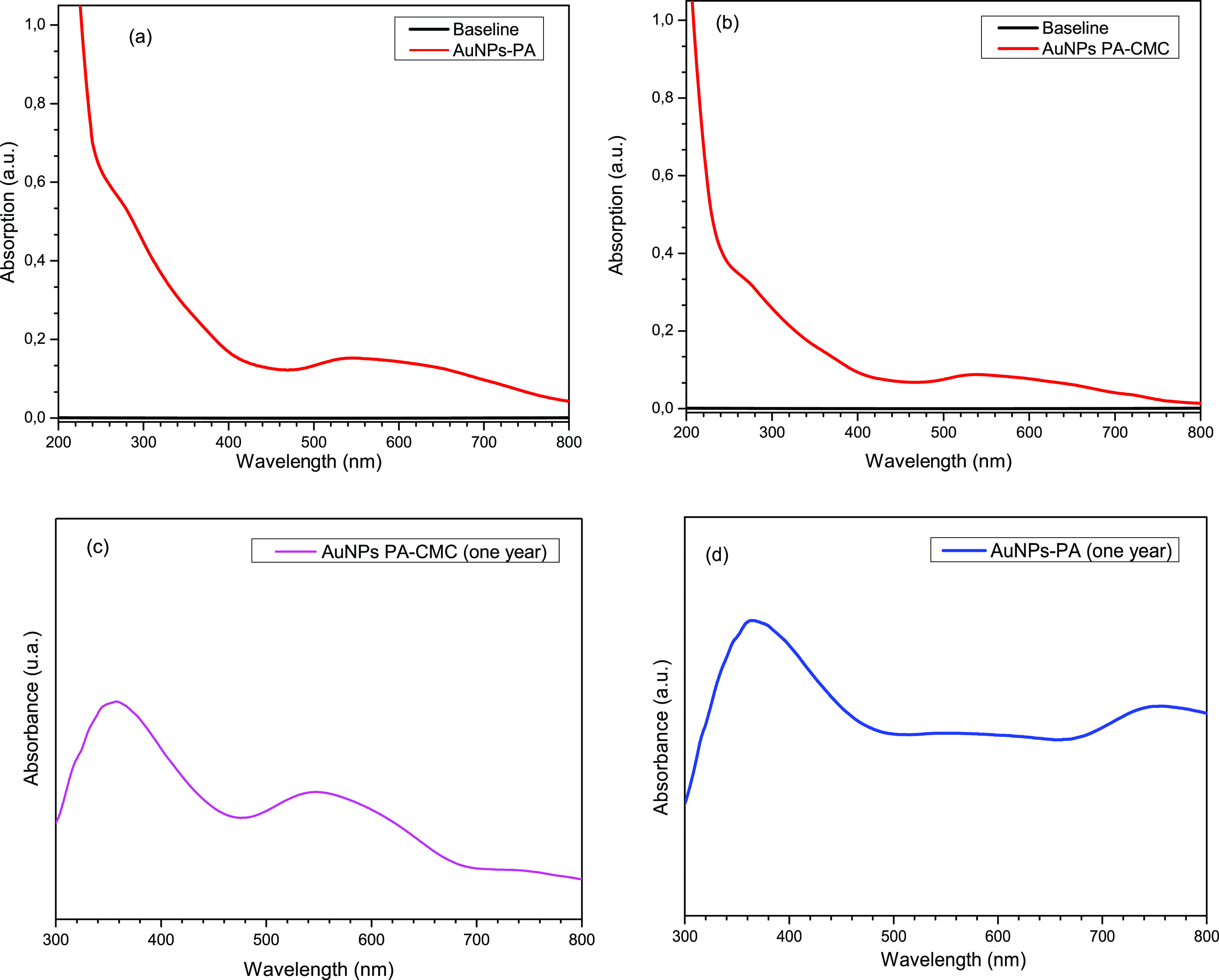
(a) UV–vis spectra of AuNPs-PA, (b) UV–vis spectra of AuNPs-PA stabilized with CMC, (c) UV–vis spectra of AuNPs-PA stabilized with CMC and obtained one year after synthesis, and (d) UV–vis spectra of AuNPs-GS without CMC one year after synthesis.

Figure 2a shows the UV–vis spectra of AuNPs-PA. In this figure, a shoulder around 277 nm attributed to the phytocomponent narcissin^36,37^ is observed, and a band centered around 550 nm is associated with the LSPR band of AuNPs-PA. Figure 2b shows the UV–vis spectra of CMC-stabilized AuNPs-PA. This figure shows that the optical absorption bands of the PA extract and of the AuNPs-PA are not modified by the presence of CMC, achieving fixation of the surface plasmon band. This result is essential since, in this synthesis technique, it is typical for the metallic nanoparticles to agglomerate and precipitate due to the low stabilizing properties offered by plant extracts and the degradation of the phytocomponents over time. Figure 2c,d shows the UV–vis spectra of the AuNPs-GS samples with CMC and without CMC, respectively, taken one year after synthesis. Figure 2c indicates that the LSPR band of AuNPs-PA is maintained; therefore, CMC prevented the aggregation and precipitation of the AuNP colloid. As in Figure 1d, Figure 2d shows a decrease in the intensity of the peaks, accompanied by a redshift in the spectrum, which can occur due to the aggregation of AuNPs.

### 3.1 TEM Microscopy

**Figure 3:**
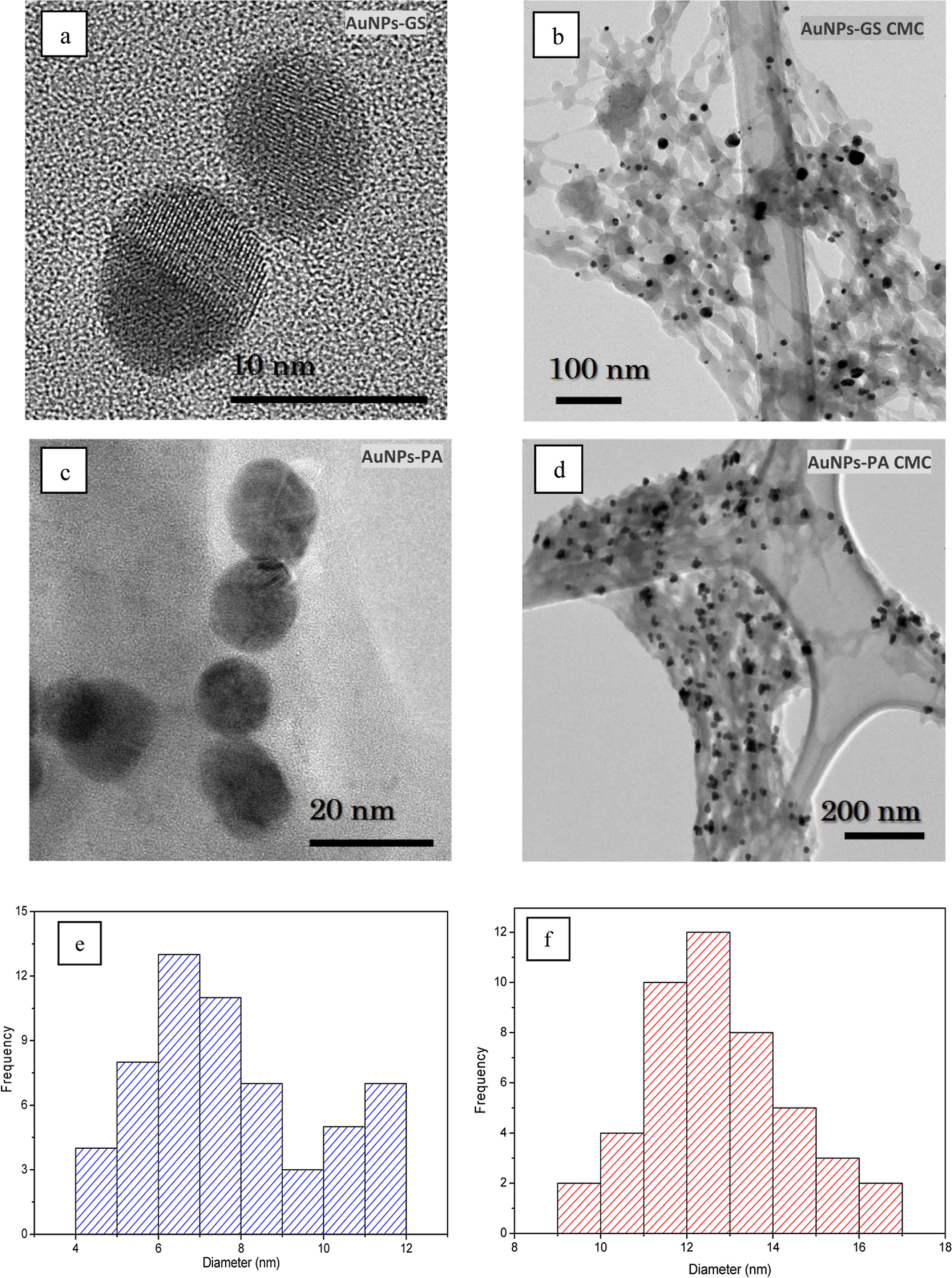
TEM images of AuNPs. (a) AuNPs-GS, (b) AuNPs-GS shows the matrix effect of CMC, (c) AuNPs-PA, and (d) AuNPs-PA shows the matrix effect of CMC. Histogram of the size distribution of (e) AuNPs-GS and (f) AuNPs-PA.

Figure 3a,c shows TEM microscopy of the AuNPs. According to these micrographs, the observed AuNPs present relatively spherical morphologies. The crystal planes in Figure 3a are evident, and the size distribution histograms present mean particle diameters of ≈7.74 and ≈12.7 nm for AuNPs-GS and AuNPs-PA, respectively.

**Scheme 3:**
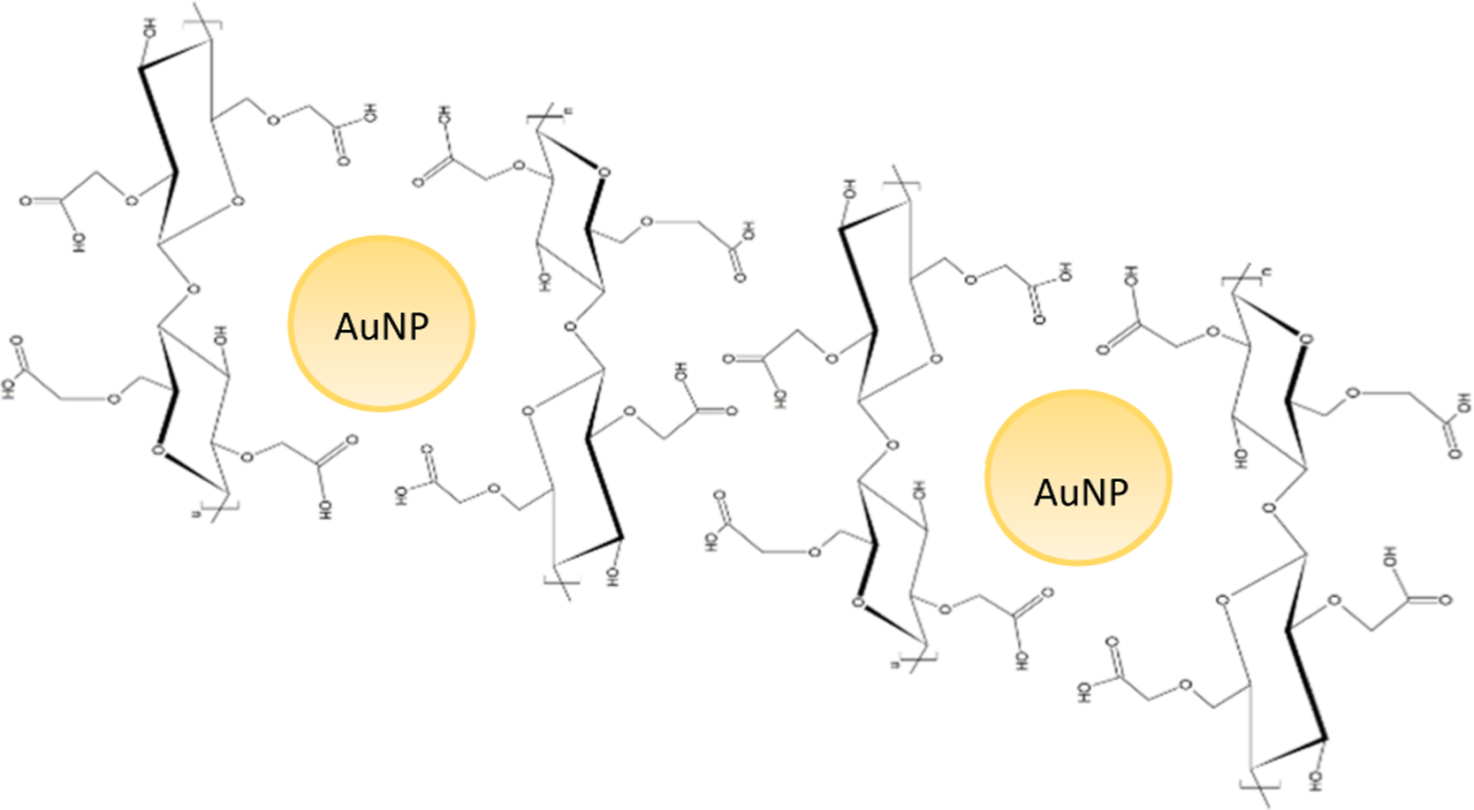
Schematic Representation of AuNPs-CMC Interactions

Figure 3b (GS) and Figure 3d (PA) show the matrix effect of CMC. According to these, the polymeric chain mixes with the AuNP colloid (dark dots), supports the nanoparticles, stabilizes them, and prevents their aggregation, allowing the AuNP colloid to last over time, allowing its conservation, and thus fixing or establishing applications of AuNPs. Scheme 3 presents a molecular schematic of the possible reaction mechanism between the molecular chain and the AuNPs.

### 3.2 SERS Analysis

**Figure 4:**
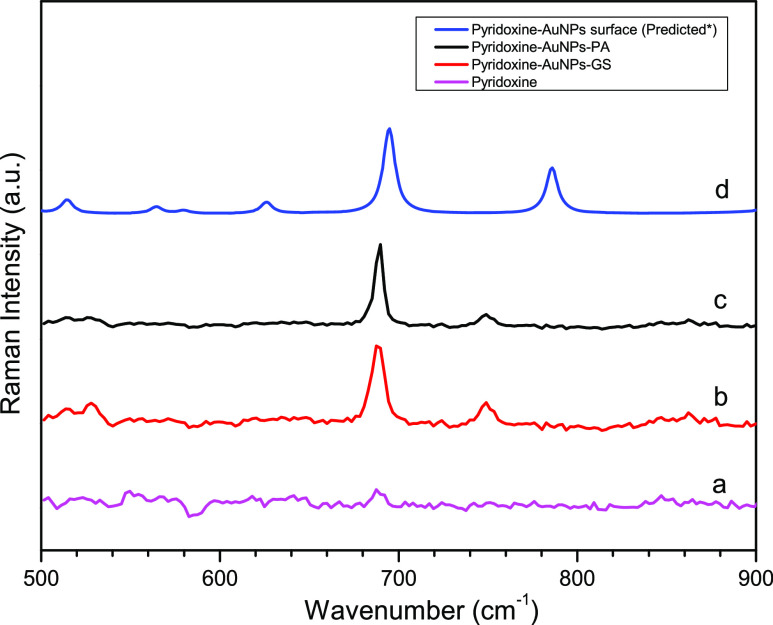
Raman spectra of Pd (a) and surface-enhanced Raman spectroscopy: Pd-AuNPs-GS (b) and AuNPs-PA (c) and Raman spectra calculated (d) with the LSDA functional.

The Pd molecule is composed of 23 atoms and 63 normal modes of vibration distributed as follows: 23 modes of stretching, 8 modes of torsion, 3 modes of deformation, 8 modes of plane deformation, 8 modes of nondeformation flat, 5 modes of angular flexion, 2 rocking modes, 2 scissors, and 4 rocking.^38,39^Figure 4 shows the Raman spectra of Pd (magenta) and Pd in AuNP nanosubstrates obtained in green synthesis with extracts from GS (red) and PA (black) plants and the surface-enhanced Raman spectra of Pd calculated. These spectra show the intensification of the Raman bands at 688 cm^–1^ (AuNPs-PA), corresponding to the lowest frequency mode of one of the six stretching modes of the pyridine ring.

**Scheme 4:**
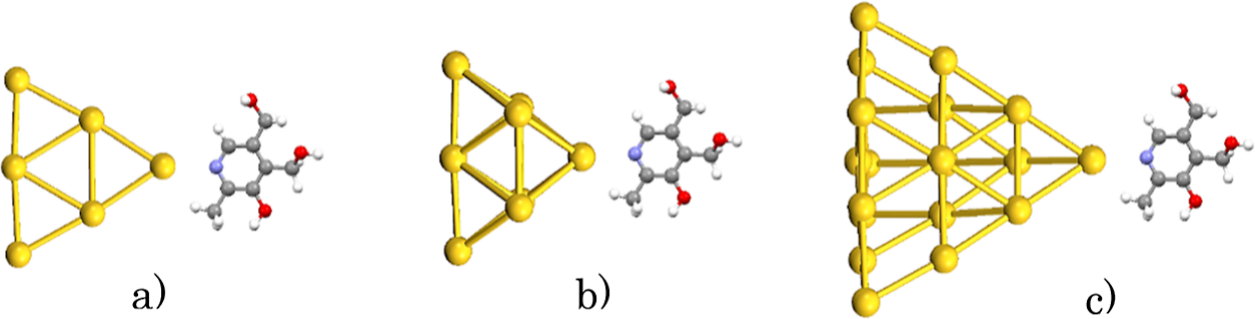
DFT Clusters of Gold in Interaction with Pd: (a) Au_6_, (b) Au_8_, and (c) Au_20_

DFT was used to obtain evidence to address the observed experimental behavior. In this sense, this type of calculation provides structural, electronic, and vibrational analyses of the interaction between systems. For this, we considered small gold species (Au clusters) with planar, solid, and hollow morphologies (Au_6_, Au_8_, and Au_20_, respectively) to study the interaction with the Pd molecule, as shown in Scheme 4.

In all the cases analyzed, a minimum local energy was found when the interaction distance between the pyridine nitrogen atom and the nearest gold atom was located between 2.08 and 2.1 Å. Similarly, an affinity for gold atoms located in the vertices was observed.^40^ Additionally, the adsorption energy was considered. This is defined as the energy required or released when a species is adsorbed on a surface and can be obtained considering the energies of the individual systems and the complex system based on the following expression
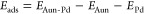
1where *E*_Aun-Pd_ is the local minimum energy obtained from the interacting system and *E*_Au_ and *E*_Pd_ represent the energy of the Au_*n*_ gold clusters (n = 6, 8, and 20) and the local minimum energy of the Pd molecule, respectively.

In addition, negative *E*_ads_ values were obtained for the cases analyzed, revealing an exothermic process, as shown in Table 1. This indicates that the interaction sites found present higher stability compared to other gold atoms on the surface.^41^

**Table 1:** Molecular Descriptors of Au-Cluster Pd at the LSDA, B3PW91, and HCTH Approximation Levels in Combination with the LANL2DZ Basis Set

DFT-functional	molecular system	morphology	interaction distance (Å)	HOMO (eV)	LUMO (eV)	*E*_ads_ (eV)	Δ*N*	RBM (cm^–1^)	other results (cm^–1^)
LSDA	Au_6_–Pd	planar	2.09116	–7.7 × 10–^3^	–5.1 × 10–^3^	–2.5 × 10–^3^	0.00335324	726.54	740^42^
	Au_8_–Pd	solid	2.08418	–7.4 × 10–^3^	–5.6 × 10–^3^	–2.4 × 10–^3^	0.00272284	725.89	
	Au_20_–Pd	hollow	2.10939	–8.0 × 10–^3^	–5.9 × 10–^3^	–2.5 × 10–^3^	0.00302208	694.74	
	Pd							711.71	
B3PW91	Au_6_–Pd	planar	2.18727	–7.7 × 10–^3^	–3.4 × 10–^3^	–1.3 × 10–^3^	0.0040236	719.96	690^43^
	Au_8_–Pd	solid	2.18104	–6.9 × 10–^3^	–3.8 × 10–^3^	–1.4 × 10–^3^	0.00214166	723.16	
	Au_20_–Pd	hollow	2.30127	–7.6 × 10–^3^	–4.3 × 10–^3^	–0.7 × 10–^3^	0.00309888	694.22	
	Pd							710.83	
HCTH	Au_6_–Pd	planar	2.24582	–7.0 × 10–^3^	–4.2 × 10–^3^	–1.0 × 10–^3^	0.0029005	727.08	692^44^
	Au_8_–Pd	solid	2.22566	–7.0 × 10–^3^	–4.6 × 10–^3^	–1.5 × 10–^3^	0.00174822	689.8	
	Au_20_–Pd	hollow	2.58395	–7.5 × 10–^3^	–4.9 × 10–^3^	–0.8 × 10–^3^	0.00227094	678.74	
	Pd							696.11	

An important parameter is the electron transfer fraction (Δ*N*); this parameter is related to the charge transfer between systems. In addition, it depends on the electronegativity and the global hardness and is represented by the Pearson method
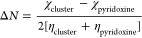
2where χ_cluster_ and η_cluster_ are the electronegativity and global hardness parameters for the cluster, respectively. χ_pyridoxine_ and η_pyridoxine_ are the parameters for the molecule, respectively.
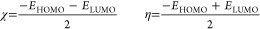
3

The values for Δ*N* are positive and oscillate between 0.002 and 0.004; this indicates that the charge transfer between both systems is favorable and may be responsible for the SERS effect if the contribution is considered under the framework of the chemical enhancement mechanism. DFT attributed a more significant intensification of Pd’s radial breathing mode (RBM) after the interaction with the Au_*n*_ clusters and predicted slight shifts. For the theoretical Pd spectrum, this mode is located at 711 cm^–1^ (Supporting Information). Other authors using the approximation level B3LYP have located a characteristic vibrational mode of Pd close to 690 cm^–1^.^38^

However, it was located between 678.7 and 727.0 cm^–1^ after interacting with the Au_*n*_ clusters under the DFT functionals used in this work. The Raman spectrum that presented the most accurate approach for the Au_20_–Pd interaction was the LSDA level of approximation, shown in Figure 4 (blue color), and it reveals essential data for the vibrational behavior. The prediction of the Raman spectrum for the Pd, Au_6_–Pd, and Au_8_–Pd cases is included in the Supporting Information. Experimentally, in this work, the RBM is located at 688 cm^–1^ after interacting with AuNPs, as shown in Figure 4. Additionally, Raman bands associated with the pyridinic ring in B-complex vitamins are located near 740 cm^–1^ after interaction with AuNPs.^42^ Furthermore, recent studies show Raman bands susceptible to electrochemical surface-enhanced Raman scattering (EC-SERS) located at 690 cm^–1^.^43^ On the other hand, semiempirical studies indicate a band associated with the pyridinic ring in vitamin B6 centered at 692 cm^–1^.^44^

The RBM in pyridinic ring molecules is a susceptible mode for the SERS effect. The shifts in the vibrational modes and intensities reflect the modification of the chemical environment around the adsorbed molecule. That is, the favorable Δ*N* and the enhancement in the Pd RBM after interaction with the Au_*n*_ clusters suggest that there is a significant chemical influence on the SERS effect observed in this system.

## 4 Conclusions

In this research, the green synthesis of gold nanoparticles was carried out. The gold nanoparticles were synthesized with the extract of the leaves of *G. sepium* and *P. alliacea*. The optical absorption spectra of the synthesized nanoparticles revealed absorption bands for the nanoparticles around 530 nm. The phytocomponents coumarin in GS and narcissin in PA are mainly responsible for the reduction of metal ions. The optical absorption spectra of the AuNP samples show that CMC does not produce additional bands of those already existing in the sample, and the LSPR bands do not show blueshift or redshift; therefore, it is considered that in this synthesis, CMC produces a matrix or surrounding effect of the nanomaterial preventing aggregation. The TEM micrographs reveal relatively spherical nanoparticles with average diameters of ≈7.7 and ≈12.7 nm for AuNPs-GS and AuNPs-PA, respectively. Also, in the micrographs, it is possible to appreciate the crystalline planes of the nanoparticles and the matrix effect of CMC on the AuNPs. The SERS analysis shows enhancements of two different vibrational bands of the Pd molecule. The Raman band, enhanced at 688 cm^–1^ by the AuNPs-PA platform, was attributed to the pyridine ring stretching mode. Complementarily, the DFT functionals employed attributed a more significant intensification of the Pd RBM mode after interaction with the Au_*n*_ groups and predicted slight changes. In the theoretical spectrum of Pd, this mode is located between 696 and 711 cm^–1^, while in the experimental study carried out here, it is located between 694.7 and 726.5 cm^–1^ after the interaction with the Au_*n*_ clusters.

## Supporting Information Available

Predicted surface-enhanced Raman spectra of Pd, Pd-Au_6_, and Pd-Au_8_ (PDF)
