# Supplementary material for: Influence of Carboxymethyl Cellulose on the Green Synthesis of Gold Nanoparticles Using Gliricidia sepium and Petiveria alliacea Extracts: Surface-Enhanced Raman Scattering Effect Evaluation
Source: ACS Omega. 2023 Nov 29;8(49):46466–74. doi: 10.1021/acsomega.3c03813 (PMC10720281; doi:10.1021/acsomega.3c03813)
Supplement: Supplementary file 1 — ao3c03813_si_001.pdf [file ao3c03813_si_001.pdf]

Influence of carboxymethyl cellulose on the green synthesis of gold nanoparticles using *Gliricidia sepium* and *Petiveria alliacea* extracts: SERS effect evaluation

Sindi Horta-Piñeres
Laboratorio de Óptica e Informática, Universidad Popular del Cesar, Apdo. Postal 200001, Valledupar, Cesar, Colombia

M. Cortez-Valadez

Departamento de Investigación en Física, Universidad de Sonora, Apdo. Postal 5-88, 83190 Hermosillo, Sonora, México.
CONACYT-Departamento de Investigación en Física, Universidad de Sonora, Apdo. Postal 5-88, 83190 Hermosillo, Sonora, México.

Duber A Ávila
Laboratorio de Óptica e Informática, Universidad Popular del Cesar, Apdo. Postal 200001, Valledupar, Cesar, Colombia.

Jesús Eduardo Leal-Pérez
Facultad de Ingeniería Mochis, Universidad Autónoma de Sinaloa, Los Mochis C.P. 81223, México.

Cesar Cutberto Leyva-Porras
Centro de Investigaciones en Materiales Avanzados S.C. (CIMAV), Chihuahua, México.

Mario Flores-Acosta
Departamento de Investigación en Física, Universidad de Sonora, Apdo. Postal 5-88, 83190 Hermosillo, Sonora, México.

Cesar O Torres
Laboratorio de Óptica e Informática, Universidad Popular del Cesar, Apdo. Postal 200001, Valledupar, Cesar, Colombia.

Supplemental information

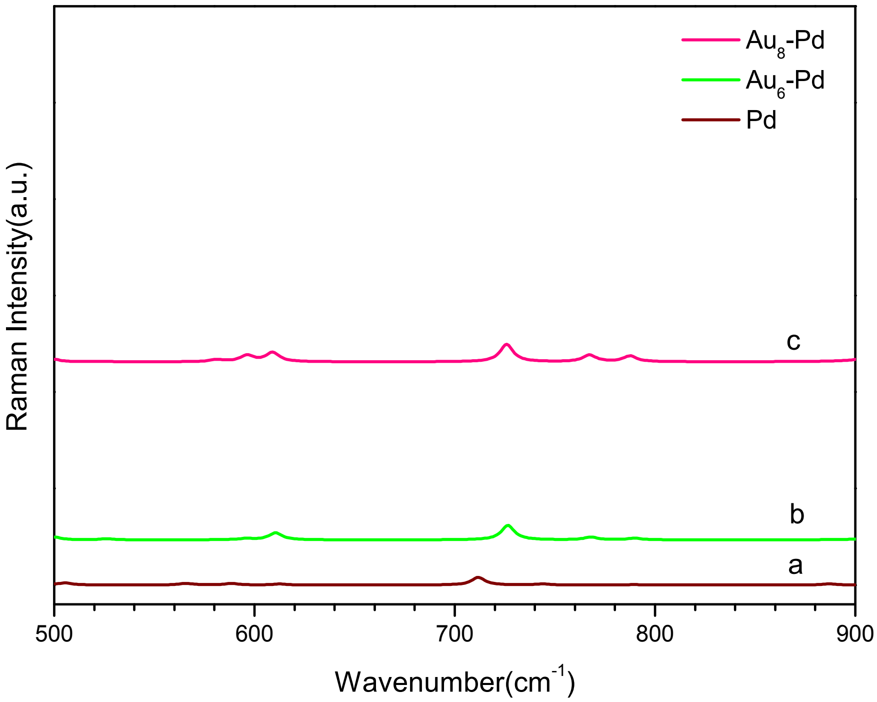

Figure S1: Predicted surface-enhanced Raman spectra of **a** pyridoxine, **b** Pyridoxine-Au_6 (green), and, **c** Pyridoxine-Au_8 (pink). *LSDA/LANL2DZ
